# Availability of substance abuse treatment services in Spanish: A GIS analysis of Latino communities in Los Angeles County, California

**DOI:** 10.1186/1747-597X-6-21

**Published:** 2011-08-12

**Authors:** Erick G Guerrero, Karen B Pan, Andrew Curtis, Erica L Lizano

**Affiliations:** 1 School of Social Work, University of Southern California, Los Angeles, USA; 2 School of Policy, Planning and Development, University of Southern California, Los Angeles, USA; 3 Department of American Studies & Ethnicity, College of Letters, Arts and Sciences, University of Southern California, Los Angeles, USA

## Abstract

**Background:**

The percentage of Latino clients entering outpatient substance abuse treatment (OSAT) in the United States has increased significantly in the past 10 years. Evidence suggests that a lack of services in Spanish is a significant barrier to treatment access among Latinos.

**Methods:**

Using a geographic information system (GIS) approach, data from the U.S. Census Bureau and the National Survey of Substance Abuse Treatment Services (N-SSATS) were analyzed to determine the geographic distance between OSAT facilities with services in Spanish and Latino communities throughout Los Angeles County, CA. Data from N-SSATS were also analyzed using logistic regression models to examine organizational characteristics and their association with offering services in Spanish. Our GIS methods are tested in their ability to provide baseline measures to inform future analysis comparing changes in demography and service infrastructure.

**Results:**

GIS analysis revealed cold spots representing high-density Latino communities with extensive travel distance to facilities offering services in Spanish. The average linear distance between Latino communities and facilities offering Spanish-language services ranged from 2 to 6 miles, while the location of the cold spots pointed to a need for services in Spanish in a particular subregion of the county. Further, secondary data analysis revealed that, on average, being privately owned (*OR *= .23, 95% CI = 0.06-0.90) was associated with a lower likelihood of providing services in Spanish compared to public facilities. Additionally, a facility with a state license (*OR *= 2.08, 95% CI = 1.12-3.88) or a higher number of Medicaid recipients (*OR *= 2.98, 95% CI = 1.76-5.05) was twice as likely to offer services in Spanish.

**Conclusion:**

Despite the significant presence of Latinos in L.A. County in 2000, low capacity was found in discrete Latino communities in terms of offering OSAT services in Spanish. Funding and regulation play a significant role in facilities' capacity to offer these services. Future studies should build from our multi-method approach to compare changes in population demography and system infrastructure and inform health care policy that seeks to improve providers' capacity to provide linguistically competent care.

## Background

Improving the accessibility of outpatient substance abuse treatment (OSAT) enhances the ability of individuals to address their drug abuse issues. Significant evidence suggests that race, ethnicity, income, and language can act as barriers to health care service access [1,2]. In the United States, Latinos are more likely to live in low-income communities with greater access to alcohol and illegal substances [3,4], and often have limited access to behavioral health services in Spanish [5]. Before addressing issues pertaining to quality of services for this largely bilingual population, it is crucial to first determine the availability of OSAT services within Latino communities.

Through the use of geographic information system (GIS) analysis, this study seeks to identify the spatial impediment between low-income Latino populations with mostly limited English proficiency and outpatient substance abuse treatment services offered in Spanish throughout Los Angeles County, California. The County of Los Angeles has the largest Latino population (4.7 million) [6] and one of largest publicly funded substance abuse treatment systems in the nation [7]. This system provides services to one of the most ethnically diverse populations in the country, yet about 42% of the total population attending treatment programs is of Latino descent, with an estimated 75% of that subset identifying as Mexican or Mexican American [8,9]. Latinos of Mexican descent served by publicly funded facilities in California are most commonly users of methamphetamines and alcohol [10]. Studies using national samples suggest that Mexican Americans report the highest rates of alcohol binging behavior compared to African Americans and whites [11,12], while Mexican American men in particular report alcohol dependence rates higher than that of males in the general U.S. population [11].

U.S. federal and state legislation, through the Patient Protection and Affordable Care Act and California's access-to-services law, mandates that all health care providers offer translation or appropriate language services to those with limited English proficiency (LEP) [13]. Despite significant representation of Spanish-speaking Latinos in the state, offering OSAT services in Spanish is not standard practice. Approximately 60% of the 1,725 facilities statewide reported having bilingual (Spanish/English) counselors, while in L.A. County, where Latinos are the largest ethnic group, 66% of the 457 treatment facilities reported offering this service [14]. Though the number of OSAT facilities may be adequate to address the unmet need in L.A. County, facilities offering services in Spanish may not be strategically located where they are needed most. This study's primary aim is to highlight potential problems of geographic inaccessibility to Spanish-language treatment services and to identify organizational factors associated with the provision of such services. A secondary aim is to inform methodologies to examine the OSAT system's capacity to improve access for a Spanish speaking client population.

### Distance and Access to Outpatient Treatment Services

The disparity between the need for and access to OSAT treatment services among ethnic minorities is significant [10,12,15,16]. The availability of substance abuse treatment services provided in minority communities has been found to differ from nonminority communities [17], and tend to be less likely to be matched or tailored to the cultural needs of minority clients [18].

Latinos are more likely than other ethnic minority groups to experience delays in accessing treatment [16,19] and receive less adequate services [18]. These two factors are associated with a low level of client satisfaction [16] and some of the most common barriers to behavioral health services among Latinos [20].

Few, if any, studies regarding Latinos and access to OSAT services have focused on distance as a potential barrier, yet research suggests that it is an important factor when considering access and completion/attrition rates [21-23]. Using a geographic information system approach, different studies have examined the distribution of treatment centers and the relationship between distance and treatment outcomes. Selecting a 15-mile radius to reflect OSAT service catchment areas in urban regions across the United States, Perron, Gillespie, Alexander-Eitzman, and Delva [24] identified urban areas considered underserved. The study found that less than 5% of California's urban areas are underserved in terms of OSAT services, despite having one of the largest urban populations in the country. Yet, the geographic distance between low-income communities and OSAT facilities offering linguistically tailored treatment services remains unknown.

Increasing evidence suggests that distance, which can impact travel times to outpatient treatment settings, can have a significant effect on OSAT service utilization. Fortney et al. [22] studied 106 clients receiving treatment for depression and found that increased travel time from providers was significantly associated with making fewer visits and a greater likelihood of receiving less effective care [22]. Similarly, Beardsley et al. [21] focused on the distance traveled by 1,735 clients to various outpatient treatment programs in an urban setting. They found that distance is strongly correlated with treatment completion and higher retention rates; specifically, clients who traveled less than one mile (less than1.6 kilometers) were more likely to complete treatment than those who traveled farther [21]. This study's larger sample size allows us to apply these findings to other urban areas, such as Los Angeles. Because Latinos report high rates of logistical barriers to receipt of services [20], they are particularly vulnerable to the effects of distance on accessing services and treatment completion.

### Increasing Access and Improving Outcomes among Latinos by offering Services in Spanish

The relationship between access to responsive services and treatment completion rates among Latinos points to a serious need for greater geographic proximity to Spanish-language services for this population. Although testing treatment outcomes is not the focus of this paper, it should be noted that studies suggest that linguistic preferences significantly impact the treatment process among Latinos, indirectly contributing to treatment outcomes [5,19,25,26]. In particular, engaging clients in their native language during the intake process increases treatment retention and compliance, which are highly associated with treatment completion and improvements in posttreatment drug use. Similarly, studies have found that limited availability of bilingual treatment services is highly associated with high attrition rates from substance abuse treatment among Latinos when compared to other racial/ethnic groups [27-30].

Highlighting potential barriers to health care access, such as distance to treatment, is of importance as past studies indicate that treatment completion rates are affected by transportation issues related to distance to outpatient treatment sources [31]. In particular, low-income individuals with significant transportation and communication challenges would be at a considerable disadvantage in terms of addressing their substance abuse issues. Given the importance of geographic proximity of Spanish-language OSAT services for Latinos with LEP, we expect that outpatient facilities located closer to communities in L.A. County with large Latino populations will be more likely to offer services in Spanish.

Organizational characteristics play a significant role in facilitating access by offering culturally and linguistically responsive services [32], particularly in racially diverse communities [33]. Access to public funding resources and state regulations influence the capacity of OSAT facilities to offer culturally responsive services in low-income minority communities [33-36]. The present study aims to answer two central research questions: (1) How geographically accessible are OSAT facilities offering services in Spanish to Latino communities? (2) What organizational characteristics serve as predictors of OSAT facilities offering service in Spanish? Based on our review of the literature, we hypothesize the following: OSAT facilities located closer to communities with large Latino populations, as well as facilities with access to government funding and regulation, will be more likely to provide Spanish-speaking services than facilities located in ethnically heterogeneous communities and not funded or regulated by government.

## Methods

### Sampling Frame

We used two sources of secondary data to map OSAT service availability and identify providers' organizational structure. The 2000 U.S. Census Bureau data [37] provided information about Latino representation in Los Angeles County. The list of OSAT facilities was retrieved from the Substance Abuse and Mental Health Services Administration's (SAMHSA) online facility locator in November 2010. In addition, data collected in 2007 from the National Survey on Substance Abuse Treatment Services (N-SSATS) yielded the organizational characteristics of OSAT facilities in L.A. County, including whether or not Spanish-language services are available at each facility. The N-SSATS data comes from SAMHSA's annual census of drug treatment facilities. Although collected yearly, these data are cross-sectional and provide information about the structural characteristics of facilities as well as client characteristics. More information about the sampling frame is available from SAMHSA [14].

### Selection Procedure

To systematically identify and compare OSAT facilities with *similar *organizational structure, three selection criteria were used when searching the SAMHSA database. Facilities were selected for inclusion if the facility was located within L.A. County and met the following criteria: (1) the facility is primarily a substance abuse treatment facility (excluding all general or mental health facilities); (2) the facility provides mainly OSAT services; and (3) the facility provides services in Spanish through a bilingual counselor.

### Analytical Framework

Information collected from both the 2000 U.S. Census and SAMHSA's facility locator in 2010 was consolidated and initially analyzed using ArcGIS 9.3 software. ArcGIS 9.3 is a mapping software system designed to facilitate the collection, management, and analysis of spatially referenced information and associated attribute data [38]. The spatial analysis used these two sources of data to map three layers: existing facilities, the availability of Spanish-language services at each facility, and the population density of Latino communities throughout Los Angeles County.

### Mapping Latino Communities

A GIS approach was used to map the distribution of Latino residents in Los Angeles County drawn from 2000 U.S. Census Bureau data. The use of census block data, the smallest unit available, allowed the finished maps to more accurately reflect actual population distributions. Final maps are displayed either using graduated colors for the entire block area or each center point.

### Locating Substance Abuse Treatment Facilities

The straight line distance was calculated between every census block in Los Angeles County and both the closest OSAT facility and the closest facility offering services in Spanish. This was achieved by calculating the linear distance between the center point of each block and the facility address extracted from SAMHSA's facility locator database. Although straight-distance measures have a central limitation--namely, less accuracy in measuring distance when compared to road network distance analysis--they have been used in other studies examining distance to a treatment facility [21,24]. While straight distance does not take into consideration the travel route and the nature of the terrain, which may impact travel time, it allows us to (1) identify regions with unmet service needs, (2) compare the different distances between these regions and the closest Latino communities, and (3) visualize areas of services with greater capacity (clusters) to provide services in Spanish. In short, while linear distance provides a conservative assessment of accessibility barriers, it is an appropriate methodology, along with logistic regression methods, to examine the OSAT system's capacity to provide Spanish-language services.

Using this approach, a total sample of 272 general OSAT facilities and a subset of 180 facilities offering services in Spanish were successfully geocoded in ArcGIS 9.3. The distance between facilities and each block center was calculated using a spatial join approach.

### Statistical Analysis

A follow-up statistical multivariate logistic regression analysis was conducted after the mapping analysis using N-SSATS data to determine which facility characteristics are associated with adoption or offering of services in Spanish. The sample for this analysis was also drawn from N-SSATS, but from a different public data depository and from a different year. We downloaded publicly available data collected in 2007 from the website of the Substance Abuse and Mental Health Data Archive (SAMHDA) at the University of Michigan's Inter-University Consortium for Political and Social Research. Although data downloaded from SAMHSA's website contained the addresses of facilities, which were required to conduct GIS analyses, SAMHDA data did not include identifiers (name, address, etc.), in order to protect their confidentiality. This sample included 383 facilities with the same characteristics (e.g., adult outpatient treatment) as those selected for the GIS analysis. Independent variables included the total number of services offered by the facility and structural factors including whether the facility was private for-profit, private nonprofit, licensed by the state, located within a hospital or certified by The Joint Commission, which is the premier professional accreditation entity in the OSAT field. All structural factor variables were dummy-coded in preparation for analysis.

Data analysis was performed using STATA/SE (Version 10) to conduct all procedures. We employed a logistic regression model with robust standard error specification for the dichotomous outcome (0 = facility does not offer services in Spanish, 1 = facility offers services in Spanish). Goodness-of-fit tests were used to determine the appropriateness of the regression model. Because N-SSATS data includes all OSAT facilities in the United States, and consequently all facilities in L.A. County, we did not need to correct for selection bias.

## Results

The following maps are presented with the eight service planning areas (SPA) that divide L.A. County into regions and are widely used by public administrators to manage issues related to service capacity. These SPAs and local regions provide the overall parameters to examine clusters of facilities offering services in Spanish and communities with higher concentrations of Latinos. In addition, in order to ease the interpretation of the maps, a kernel density surface was created within the GIS. This is a technique frequently used to find high concentrations of a variable (e.g., Latino communities, OSAT facilities); in this case, it was used to develop cold spots, which serve in this study as measures of excessive distance to a facility. Density values are not included here since these maps are intended only as a visual guide to emphasize patterns.

**Figure 1:**
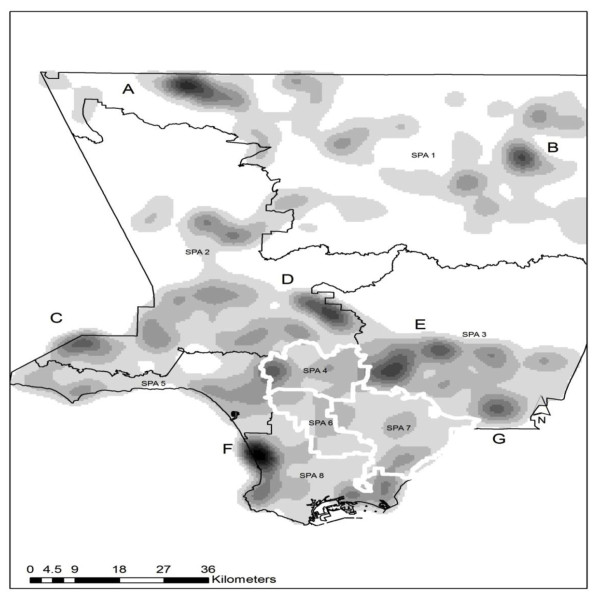
**Density in cold spots of extensive distance to Spanish-language services by SPA in L.A. County**. Cold spots showing the largest distances between census blocks and the nearest facilities providing services in Spanish are mapped as darker areas. The letters indicate general cold spots, with E (seen in Figure 2) representing the combined area of the two dark patches beneath.

In general, areas with darker concentrations have the least access to facilities with Spanish-language services. These locations may be justified by the presence of a non-Spanish-speaking population. In order to further assess the regions with the greatest discrepancy between the density of the Latino population and accessibility to Spanish-language services, seven cold spots were identified for detailed investigation (A to G in Figure 1).

**Figure 2:**
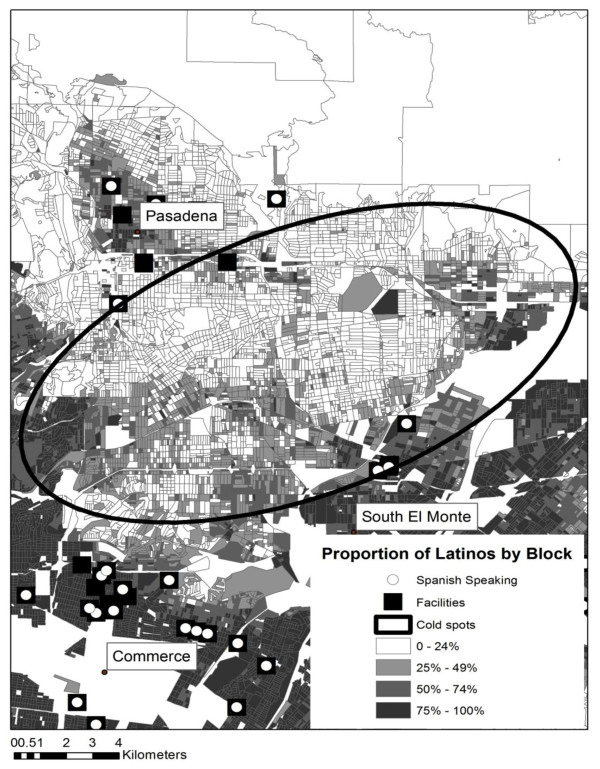
**Cold spot E and facilities offering services in Spanish**. Map shows cold spot E, which suggests an area with distance impediment to a treatment facility, overlaid on a graduated color surface of Latino density per census block.

**Figure 3:**
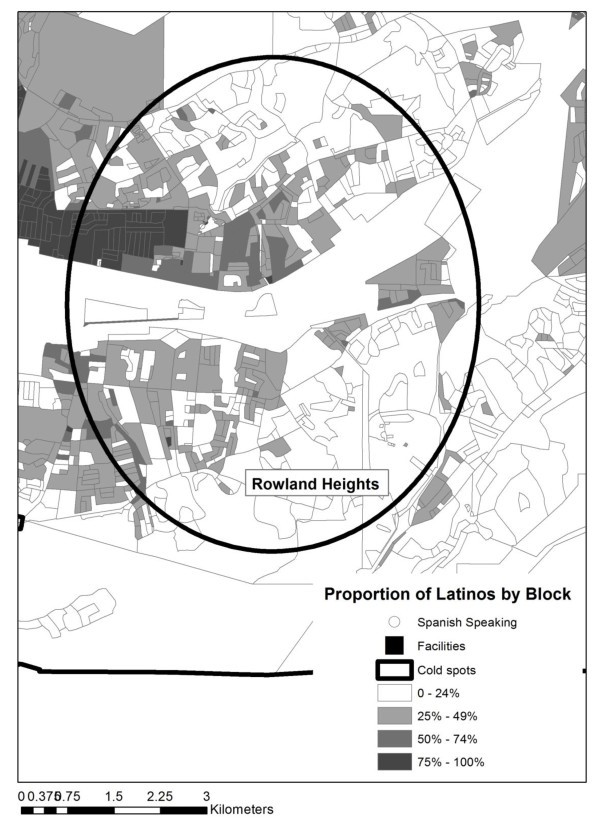
**Cold spot G and facilities offering services in Spanish**. Map shows cold spot G, which suggests an area with distance impediment to a treatment facility, overlaid on a graduated color surface of Latino density per census block.

Table 1 provides a summary of each of the cold spots represented in Figure 1. The total population, total Latino population, and number of blocks are reported for each cold spot. In addition, the percentage of Latino-dominated blocks (where greater than 50% or 75% of the population is Latino) is also shown. Table 1 used in conjunction with Figure 1 aids with the interpretation of accessibility. For example, although there appears to be less access to Spanish OSAT services in areas A to C, this is understandable given the general lack of a Spanish-speaking cohort. Access to Spanish OSAT services in Areas E to G, located in the San Gabriel Valley region, is of greatest concern, particularly for cold spot G, in which approximately one third of the population is Latino and the minimum distance to the closest OSAT facility for census blocks in which at least 50% of the population is Latino is 4 km (2.48 miles), while the farthest distance is 9 km (5.59 miles). This can be seen graphically in Figures 2 and 3 (cold spots E and G). In both maps the proportion of Latinos per census block are displayed, along with the cold spot outline and the locations of facilities (black squares) and Spanish-language facilities (black squares with a white dot).

**Table 1:** Cold spots of extensive distance to a facility and their associated Latino population

Cold Spots	Total Blocks	Total Population	Percentage Latino	L > 50%	L > 75%
A	438	1067	12	5	3
B	372	14268	33	10	2
C	449	29337	6	0	0
D	1445	113308	14	2	0
E	5112	541877	35	18	7
F	2008	132914	15	4	1
G	726	87197	35	15	6

L > 50% and L > 75% represent the percentage of blocks where more than 50% or 75% of the population is Latino.

These maps also help reveal the spatial complexity involved as the analysis moves to a finer scale. For example, there are clusters of facilities surrounding cold spot E both to the northeast and southwest, as well as three facilities along the eastern edge. No facility exists within the region, despite concentrations of Latinos who are at a distance to the closest facility that exceeds the threshold identified by Beardsley et al. (less than one mile) as resulting in poorer treatment outcomes [21]. The same is true for cold spot G; facility locations to the northwest of the cold spot are visible, and while these can be justified due to the higher concentration of Latino populations in this area, the distance between Latino concentrations within the cold spot and any facility could prove an impediment to a successful outcome.

### Statistical Analysis

#### Client and organizational characteristics

The logistic regression models highlight the significant role of client and structural characteristics in the probability that OSAT facilities will offer services in Spanish (Table 2). The most relevant findings show that facilities that were privately owned (*OR *= .23, 95% CI = 0.06-0.90) were less likely than publicly owned facilities to provide services in Spanish. In contrast, those facilities with a state license (*OR *= 2.08, 95% CI = 1.12-3.88) and those with a higher number of clients covered by Medicaid (*OR *= 2.98, 95% CI = 1.76-5.05) were two to three times more likely to provide services in Spanish compared to non-state-licensed facilities and those with fewer Medicaid clients.

**Table 2:** Multivariate logistic regression on facilities offering services in Spanish in L.A. County

	Services in Spanish	
	
	OR	**95% C. I**.
*Independent variables*		
Number of services	1.05 *	1.01 1.10
For profit^a^	0.23 *	0.06 0.90
Non-for profit^a^	0.29	0.08 1.07
Medicaid	2.98 **	1.76 5.05
State Licensed	2.08 *	1.12 3.88
TJC	1.18	0.45 3.11
Facility in hospital^b^	0.26	0.06 1.03
Constant	1.12	0.28 4.46
Observation	388	

Non-standardized parameter estimates, with robust standard errors in parentheses from two-tailed tests.

TJC = The Joint Commission

^a^Public facility is the referent

^b^Free-standing facility is the referent

*p < .05

**p < .01

### Limitations and Interpretations

Although we used the same N-SSATS survey data for the GIS and statistical regression analyses, different data collection waves were used. The statistical analysis of N-SSATS used data from 2005, while the SAMHSA facility locator offered data updated as of November 2010. Due to confidentiality issues, SAMHSA data does not have organizational characteristics while N-SSATS data has no identifying information, potentially increasing the risk of describing different samples in the GIS and regression analysis. Yet because the N-SSATS data on treatment facilities represents the universe of treatment providers, both samples used are highly correlated.

A second issue is the use of linear distance as a measure of accessibility. While measuring access to treatment accurately is essential, the current paper uses straight distance as a baseline conceptual measure to identify potential regions with unmet service needs and evaluate system capacity to offer these services. We were more concerned, however, with testing the utility of GIS methods to provide a picture of unmet service need using census population data from 2000 and services data from 2010. While greater distance to Spanish-speaking providers was identified in distinct regions, the findings should be interpreted with caution given the use of 2000 data on Latino communities and 2010 data on the OSAT system. Our multi-method analyses using 2000 data, however, suggest that the unmet need for services in Spanish is potentially even more pervasive than found here, given the prevalence and growth of Latino communities throughout L.A. County.

## Conclusions and Implications

Using a multi-method approach, we identified specific areas with limited availability of OSAT services in Spanish in the county with the largest population of Spanish-speaking Latinos in the United States. While most communities have access to services in Spanish, the northeast area of the county--representing SPA 3 with cities such as Rowland-Hacienda Heights, West Covina, La Puente, Alhambra, El Monte, and Rosemead--reported the greatest linear distance to treatment facilities offering services in Spanish. Maps of these Latino communities, which surround cold spots E and G, show the significant scarcity of general and Spanish-speaking providers. This is a geographic region that is home to almost one fifth (18%) of the county's Latino residents, and where 70% of Latino residents report speaking primarily Spanish in the home [39].

It is highly likely that the disparity between the need for Spanish-language substance abuse treatment and geographical accessibility to Spanish OSAT services in certain regions of the County (e.g., SPA 3) is greater than what is presented in this study. U.S. Census data from 2000 yield conservative Latino population estimates in L.A. County, and although final 2010 Census estimates are not yet fully available, it is evident that the Latino population has grown rapidly in the last decade. Possibly the most interesting finding extracted from these maps is that the areas traditionally known to have high Latino populations (the three highlighted SPAs--4, 6, and 7--in Figure 1) may be relatively well served. It is the more fragmented, but expanding, communities that may not be accurately depicted in data from 2000 (i.e., SPA 3) wherein the greatest need for language capacity building exists. Considering that Latinos are the fastest-growing ethnic minority group [6], the unmet service need found in this study is likely to be more pronounced in population data from 2010, as preliminary information indicates significant growth in SPA 3. When combined with the oversimplification of linear distance and considering that the most recent data are the facility locations, our findings suggest that an inaccessibility problem exists for neighborhoods in these areas. It is expected that there will always be neighborhoods that are poorly served due to isolation. However, these results, especially if overlaid with other socioeconomic measures, will make for an interesting comparison between 2000 and 2010 census population distributions in future studies.

It should also be noted that access to services as presented here does not take into account other geographic subtleties, such as transportation routes or cultural boundaries. The results presented here are intended to be illustrative and suggest further avenues of investigation rather than exhaustive. The conservative measure of distance used here, which maps the separation between census block and a facility with a lower value than if networks and other impediments are introduced, identifies areas that far exceed the threshold of poorer outcomes identified by Beardsley et al. (less than one mile) [21]. It should be noted that the impediment of distance would vary by geographic location, with differences between urban and rural locales, and between different urban areas. Los Angeles County is a densely populated landscape with economic, transportation, cultural, and ethnic challenges. It is for this reason that the threshold identified by Beardsley et al. [21] for Baltimore, Maryland, was deemed appropriate as a comparison population.

In this study we were able to respond to our question about OSAT Spanish service availability and program characteristics, revealing areas with a high need to develop service capacity. Offering efficient OSAT services in Spanish, however, goes beyond locating facilities closer to Latino communities. It involves developing and implementing program capacity to respond to the needs of the nearby community, which is most likely to utilize these resources. Consistent with other studies, our logistic regression models suggest that developing capacity to offer linguistically tailored services requires support from government resources and licensing regulation [34-36,40]. In particular, it is important to encourage for-profit and hospital providers to offer linguistically tailored services, especially given the emphasis on access in recent health care legislation.

The fact that Los Angeles County is the home to one of the largest Latino communities in the United States, coupled with a need for substance abuse services in Spanish [10], makes it highly important for the largest publicly funded OSAT system to comply with federal and state laws by offering culturally and linguistically responsive services. As health care reform may generate greater access to services for millions of Spanish-speaking people who lack health insurance, health care management policy should focus on building capacity in L.A. County to offer evidence-based culturally responsive practices. These practices can include diversifying the ethnic composition of providers, improving the skills of providers to conduct outreach in Latino communities, involving community leaders and family members in continuing care plans, and assessing the English proficiency of clients to evaluate program performance.

## Competing interests

The authors declare that they have no competing interests.

## Authors' contributions

EG designed the study and conducted the statistical analysis. KP managed the literature searches and summaries of previous related work, contributed to the GIS analysis, and along with EL, helped draft the initial versions of the manuscript. AC conducted the GIS analysis and drafted the results section. All authors contributed to and have approved the final manuscript.
